# Ovarian Ectopic Pregnancy as IVF Complication: First Report in a Gestational Carrier

**DOI:** 10.1155/2018/8190805

**Published:** 2018-12-31

**Authors:** Lindsay Hasegawa, Patricia Nascu, John McNaught

**Affiliations:** ^1^Michael G. DeGroote School of Medicine, McMaster University, Hamilton, ON, Canada; ^2^Department of Obstetrics and Gynecology, Stratford General Hospital, Schulich School of Medicine & Dentistry, Western University, London, ON, Canada; ^3^MyHealth Centre, London, ON, Canada

## Abstract

Ovarian pregnancy is a rare subtype of ectopic pregnancy with an increased incidence after assisted conception. We present a 31-year-old gestational carrier who presented with suprapubic and pelvic pain at 6 weeks and 2 days' gestation. An ultrasound scan demonstrated an empty uterus and a complex mass in the left adnexa. Operative laparoscopy was performed and an ovarian pregnancy was found and treated. We believe this to be the first report of ovarian pregnancy after IVF in a gestational carrier. Appropriate counselling of surrogate mothers is of utmost importance as the risk of ectopic pregnancy is increased by using assisted reproduction technology. Although ovarian pregnancy still remains a rare event, the possibility of this condition should always be considered.

## 1. Introduction

The incidence of spontaneous primary ovarian pregnancy ranges from 1:7000 to 1:40,000 pregnancies, accounting for 3.6% of all ectopic pregnancies [1]. With the development of assisted reproductive technologies the incidence of ovarian pregnancy has been increasing. The incidence of ovarian pregnancy after IVF-ET is reported to be 0.3% of all IVF pregnancies and 6% of all IVF ectopic pregnancies [2, 3]. There have been a few reported cases of primary ovarian pregnancy following assisted reproductive technologies such as IVF, ICSI, and GIFT. There are reports of occurrence with fresh, cryopreserved, and donor embryos. To our knowledge, there have been two reports of ovarian pregnancy with donor embryos, but we believe this is the first case in a gestational carrier [4, 5]. The mechanisms behind ovarian pregnancy are not completely understood; however, two distinct mechanisms have been established: direct fertilization inside the ovary and ectopic implantation of a fertilized embryo with retrograde migration from the endometrial cavity to the ovarian surface [6]. The latter would be the mechanism in cases occurring after ART. The gold standard of treatment is considered laparoscopic surgery.

## 2. The Case

A 31-year-old gravida 2, para 1 woman presented to the emergency department with sudden onset suprapubic and pelvic pain. The patient was a gestational carrier and she had undergone day 5 fresh blastocyst transfer after IVF during a spontaneous cycle. She was otherwise healthy and her only medication at the time of presentation was micronized 17B- estradiol (ESTRACE) 2mg orally TID.

At the time of her presentation she was posttransfer day 25, correlating with an estimated gestational age of 6+2wks. Upon clinical assessment in the emergency department the patient reported intermittent suprapubic and pelvic pain beginning the previous day. She denied any syncopal episodes, bowel or bladder symptoms, or vaginal bleeding. Investigations revealed a beta-hCG of 1038IU/L, Rh negative status with no antibodies, and hemoglobin of 140 g/L. Ultrasound reported a retroverted uterus with no evidence of an intrauterine pregnancy or endometrial thickening. The right ovary appeared normal; however there was a complex mass surrounding the left adnexa measuring 7.5x3.5x3.5cm. There was no distinct gestational sac seen and a moderate amount of echogenic free fluid in the pelvis. Thus, a ruptured ectopic pregnancy was suspected and a decision was made to proceed to surgical management.

The patient was brought to the OR for emergent laparoscopy under general anesthetic. Intraoperatively a moderate amount of hemorrhagic material was found in the cul-de-sac. There was active bleeding from the left ovary, which involved at least a third of the ovarian cortex, as well as the fimbria of the left fallopian tube. It was our intention to preserve the patient adnexa, and we started the surgery as a partial oophorectomy. Unfortunately the bleeding encountered was difficult to control. Given the extent of hemorrhage, a left salpingooophorectomy was performed without complications. Hemostasis was achieved and the patient was transferred to recovery in stable condition.

The patient had an uneventful postoperative recovery and was given Rh immunoglobulin prior to discharge. Pathology reported products of conception including chorionic villi in the left ovary, as well as congestion of the left fallopian tube with unremarkable mucosa, confirming the diagnosis of left ovarian pregnancy.

## 3. Discussion

This case meets the diagnostic criteria for ovarian pregnancy as described by Spiegelberg: (i) the gestational sac was located in the region of the ovary, (ii) the ectopic pregnancy was attached to the uterus by the ovarian ligament, (iii) ovarian tissue in the wall of the gestational sac was proved histologically, and (iv) the tube on the involved side was intact [7].

The proposed mechanisms behind ovarian pregnancy are direct fertilization inside the ovary and ectopic implantation of a fertilized embryo with retrograde migration from the endometrial cavity to the ovarian surface. General risk factors include pelvic inflammatory diseases, previous gynecologic surgery, tubal pathology, and IUD use. Possible explanations for the increased risk with IVF procedures include higher volume of the culture medium, a higher pressure of injection into the uterus, ovarian surface scars from oocyte retrieval, high estrogen levels, progesterone levels, ovarian hypervascularity after ovarian stimulation, a high number of transferred embryos, and transfer of blastocyst [3, 4, 6].

In this case, with the patient being a gestational carrier for an infertile couple, there were no typical risk factors. It is possible that the transferred embryo did not implant at all, and rather the patient became pregnant spontaneously. However, the patient abstained from sexual intercourse during the embryo transfer cycle, as instructed.

Laparoscopic management with resection of ovarian gestation and preservation of ovarian tissue is considered the gold standard treatment; however medical management with methotrexate has been reported [8]. It is important for clinicians and patients to appreciate the risk of primary ovarian pregnancy after ovarian suppression and donor embryo transfer. Even gestational carriers will have an increased risk of ectopic pregnancy due to the ART process, and appropriate counselling is paramount. Clinical suspicion, sonographic assessment, and close follow-up can help ensure early diagnosis and treatment and straightforward recovery.
